# Plasma concentrations of lysophosphatidic acid and the expression of its receptors in peripheral blood mononuclear cells are altered in patients with cocaine use disorders

**DOI:** 10.1038/s41398-023-02523-1

**Published:** 2023-06-21

**Authors:** María Flores-López, Nuria García-Marchena, Francisco J. Pavón-Morón, Nerea Requena-Ocaña, Laura Sánchez-Marín, Laura Martín-Chaves, Mónica García-Medina, Carmen Pedraza, Estela Castilla-Ortega, Juan J. Ruiz, Fernando Rodríguez de Fonseca, Pedro Araos, Antonia Serrano

**Affiliations:** 1 Instituto de Investigación Biomédica de Málaga y Plataforma en Nanomedicina (IBIMA-Plataforma BIONAND), 29590 Málaga, Spain; 2 Unidad de Gestión Clínica de Salud Mental, Hospital Regional Universitario de Málaga, 29010 Málaga, Spain; 3 Departamento de Psicobiología y Metodología de las Ciencias del Comportamiento, Facultad de Psicología, Universidad de Málaga, 29010 Málaga, Spain; 4 Unidad de Adicciones-Servicio de Medicina Interna, Institut d’Investigació en Ciències de la Salut Germans Trias i Pujol (IGTP), 08916 Badalona, Spain; 5 Unidad de Gestión Clínica Área del Corazón, Hospital Universitario Virgen de la Victoria de Málaga, 29010 Málaga, Spain; 6 Centro de Investigación Biomédica en Red de Enfermedades Cardiovasculares (CIBERCV), Instituto de Salud Carlos III, 28029 Madrid, Spain; 7 Centro Provincial de Drogodependencias de Málaga, Diputación Provincial de Málaga, 29010 Málaga, Spain; 8 Unidad de Gestión Clínica de Neurología, Hospital Regional Universitario de Málaga, 29010 Málaga, Spain

**Keywords:** Diagnostic markers, Addiction

## Abstract

We have recently reported alterations in the plasma concentrations of lysophosphatidic acid (LPA) in patients with substance use disorders. In order to further explore the potential role of the LPA signaling system as biomarker in cocaine use disorders (CUD) we conducted a cross-sectional study with 105 patients diagnosed with CUD and 92 healthy controls. Participants were clinically evaluated and blood samples were collected to determine plasma concentrations of total LPA and LPA species (16:0-, 18:0-, 18:1-, 18:2-, and 20:4-LPA), and the gene expression of LPA_1_ and LPA_2_ receptors in peripheral blood mononuclear cells. We found that patients with CUD had significantly lower plasma concentration of the majority of LPA species, while the mRNA expression of LPA_1_ receptor was found to be higher than controls. Moreover, we found a positive association between plasma concentration of 20:4-LPA and relevant CUD-related variables: age of onset cocaine use and length of cocaine abstinence. The statistical analysis revealed sex differences in concentrations of total LPA and LPA species, and women showed higher LPA concentrations than men. Furthermore, studies in rats of both sexes showed that plasma concentrations of total LPA were also altered after acute and chronic cocaine administration, revealing a sexual dimorphism in these effects. This study found alterations on the LPA signaling system in both, patients with CUD and rats treated with cocaine. Our results demonstrate that LPA signaling is impacted by CUD and sex, which must be taken into consideration in future studies evaluating LPA as a reliable biomarker for CUD.

## Introduction

Over the last decades, cocaine use has increased, representing a public health problem worldwide [1]. Cocaine is a psychoactive substance that directly damages the Central Nervous System (CNS) [2], and its use is associated with numerous medical pathologies [3–6]. Approximately 20% of people who start using cocaine will develop a cocaine use disorder (CUD) [7]. In addition, patients with CUD present a high prevalence of psychiatric comorbidity, including mood disorders, anxiety disorders, psychotic disorders, borderline personality disorders, and antisocial disorders [8, 9].

The co-occurrence of comorbid mental and somatic disorders in patients with CUD complicates their diagnosis, treatment and prognosis because some of these disorders have overlapping symptoms as a consequence of sharing common abnormalities in brain structure and functioning [10, 11]. Therefore, the search for objective biological tests that determine the degree of consumption, severity of CUD, toxicity, and response to treatment in patients with CUD would be useful in clinical practice. Thus, the identification of biomarkers for CUD would be fundamental in the diagnosis, stratification, prognosis, and therapeutic orientation of these patients. In this regard, relevant molecular signaling systems involved in the modulation of the response to cocaine are gaining interest to identify valid and reliable biomarkers for CUD.

Among the potential candidates, several preclinical studies have suggested a potential role of lysophosphatidic acid (LPA) in drug addiction [12–15], and other studies have linked LPA signaling to substance use disorders (SUD) and its comorbidity [16–18].

LPA is an endogenous bioactive lipid that is involved in a variety of biological processes through activation of a complex family of G protein-coupled receptors (LPA_1-6_) that are ubiquitously distributed in the CNS and peripheral tissues [19, 20]. Because the LPA signaling system is involved in brain plasticity and behavior in cerebral through interacting with relevant neurotransmitter systems (e.g., dopaminergic, glutamatergic, GABAergic and endocannabinoid neurotransmission) in cerebral areas associated with reward and memory processes, it has been suggested to be involved in drug-addiction-associated maladaptations [21]. Interestingly, LPA can be detected in many body fluids, although blood is the major source of this lipid mediator in mammals [22]. LPA is synthetized through different metabolic pathways, being the autotaxin (ATX) the primary enzyme responsible of its production [20, 23, 24]. The synthesis of LPA through the different metabolic routes results in the production of several LPA species, depending on the acyl group, that may differ in their biological actions [17, 25, 26]. Among these different chemical species, the most abundant in human blood are the 16:0-, 18:0-,18:1-, 18:2- and 20:4- acyl LPA [27].

Previous studies from our group have suggested a potential role of LPA as a reliable biomarker for SUD, mainly in patients diagnosed with alcohol use disorder (AUD) [16–18]. Therefore, LPA could be a potential biomarker for mild cognitive impairment in abstinent patients with AUD [17], as well as plasma concentrations of ATX and LPA could be a preventive biomarker of liver disease in these patients with AUD [16]. In addition to these studies, we have recently explored the plasma concentrations of LPA in patients with SUD, but considering the different substance uses, and we have found that the concentration of the species of LPA are affected by the type of SUD [18]. In fact, patients diagnosed with CUD alone or in combination with AUD display lower LPA concentrations than healthy controls or patients with AUD, suggesting that a pathological use of cocaine is associated with alterations in plasma concentration of LPA species. In addition to these clinical evidence, preclinical studies also have reported the involvement of LPA signaling in cocaine addiction models in rodents because cocaine induces long-term changes in neuroplasticity, neural connection and memory [14, 15, 21].

Based on these observations, we hypothesize that alterations induced by chronic cocaine use in LPA signaling at the systemic level may be linked to CUD. Therefore, we decided to explore whether pathological use of cocaine could lead to relevant changes in LPA signaling in both men and women, which may contribute in the identification of potential biomarkers for CUD in future studies. To this end, we determined the concentrations of LPA species in the plasma of healthy control subjects and abstinent patients with CUD who were recruited from outpatient treatment programs. In addition, we explored whether the plasma concentrations of LPA species were affected by cocaine-related variables in these patients. To a better understanding of the alterations in the LPA signaling associated to cocaine use, we also analyzed the mRNA expression of LPA_1_ and LPA_2_ receptors in peripheral blood mononuclear cells (PBMCs) of abstinent CUD patients and healthy controls. Finally, additional studies in male and female Wistars rats were performed to test the impact of cocaine administration on the total LPA concentrations in plasma, exploring the effects of different doses after an acute administration as well as the effects of the duration of the abstinence after a chronic treatment with cocaine.

## Materials and methods

### Participants and recruitment

This cross-sectional study included 197 Caucasian volunteers who were divided into two groups: (i) 105 patients diagnosed with CUD (CUD group), and (ii) 92 healthy control subjects (control group) matched by body mass index (BMI) and sex composition with the CUD group. Patients were recruited from outpatient treatment programs for cocaine at *Centro Provincial de Drogodependencias* (Málaga, Spain). The control participants were recruited from a multidisciplinary staff cohort of volunteers working at the *Hospital Regional Universitario de Málaga* (Málaga, Spain).

To be eligible for the present study, participants with CUD had to meet the following inclusion criteria: ≥18 years of age (up to 65 years) and diagnosis of lifetime CUD. The exclusion criteria included a personal history of long-term inflammatory diseases or cancer, cognitive or language limitations, pregnant or breast-feeding women, and infectious diseases. With regard the control group, the exclusion criteria also included the diagnosis of psychiatric disorders in Axis I, II and the problematic use of substances.

### Ethics statements

Each participant signed a written informed consent form after a complete description of the study. All the participants had the opportunity to express any questions or concerns. The study and protocols for recruitment were approved by the Ethics Committee of the Hospital Regional Universitario de Málaga in accordance with the Ethical Principles for Medical Research Involving Human Subjects adopted in the Declaration of Helsinki by the World Medical Association (64th WMA General Assembly, Fortaleza, Brazil, October 2013) and Recommendation No. R (97) 5 of the Committee of Ministers to Member States on the Protection of Medical Data (1997), and Spanish data protection act [Regulation (EU) 2016/679 of the European Parliament and of the Council 27 April 2016 on the protection of natural persons with regard to the processing of personal data and on the free movement of such data, and repealing Directive 95/46/EC (General Data Protection Regulation). All collected data were given a code number to guarantee privacy and confidentiality.

### Clinical assessments

All the sociodemographic and clinical data were collected form the participants by trained and experienced psychologists using different psychiatric interviews. Substance use disorders and other psychiatric disorders were diagnosed according to the DSM-IV-TR criteria using the Spanish version of the Psychiatric Research Interview for Substance and Mental Disorders (PRISM) [28]. This version of PRISM is a semi-structured interview with good psychometric properties in the evaluation of SUD and the main psychiatric comorbid disorders in a substance-addicted population [28, 29]. Healthy control subjects were evaluated using the Spanish version of the ‘Composite International Diagnostic Interview’ (CIDI) for detection of psychiatric disorders in general population [30] and the PRISM module 1 to assess sociodemographic variables.

### Blood collection

Blood samples were obtained in the morning after fasting for 8–12 h. Venous blood samples were extracted into 10 mL K_2_ EDTA tubes (BD, Franklin Lakes, NJ, USA) and immediately processed to obtain plasma and PBMCs.

#### Plasma extraction and rapid detection tests for infections

To obtain the plasma, blood samples were centrifuged at 2200 × *g* for 15 min (4 °C). All samples were individually assayed to detect infectious diseases by commercial rapid tests for HIV, hepatitis B, hepatitis C (Strasbourg, Cedex, France) and SARS-CoV-2 (Bio-Connect, Huissen, The Netherlands). The plasma samples were individually characterized, registered, and stored at −80 °C until further analyses.

#### PBMC extraction

PBMCs were isolated using Ficoll density gradient centrifugation. After creating a density gradient, blood samples were diluted with saline 1:1 and were centrifuged at 800 × *g* for 20 min (22 °C). Then, the PBMCs were removed from the white phase using a Pasteur pipette and were washed with saline to remove any remaining platelets. Finally, PBMCs were individually characterized, registered, and stored at −80 °C until further analyses.

### Analysis of LPA species

Plasma concentrations of five LPA species [1-palmitoyl-LPA (16:0-LPA), 1-stearoyl-LPA (18:0-LPA), 1-oleoyl-LPA (18:1-LPA), 1-linoleoyl-LPA (18:2-LPA) and 1-arachidonoyl-LPA (20:4-LPA)] were determined using a liquid chromatography with the tandem mass spectrometry (LC-MS/MS) method as previously described [17, 18]. Specifically, the detection of LPA species was performed using an ACQUITY UPLC system (Waters Associates, Milford, MA, USA) for chromatographic separation and a Xevo TQ-S micro triple quadrupole mass spectrometer (Waters Associates, Milford, MA, USA) with an orthogonal Z-spray-electrospray interface (ESI). Data management was performed with The TargetLynx XS application/option in the Waters MassLynx Software v4.1. Plasma concentrations of LPA species were expressed as nanograms of protein per milliliter of plasma (ng/mL) and LPA total was calculated by adding the concentrations of the measured LPA species [16–18, 27].

### RNA isolation from PBMCs and RT-pPCR analysis

Real-time PCR was used to quantify the relative mRNA levels of LPA_1_ (*LPAR1*) and LPA_2_ (*LPAR2*) receptors. Total RNA was extracted from PBMC samples using Trizol Reagent (Gibco BRL Life Technologies, Baltimore, MD, USA) and the concentrations were quantified using a spectrophotometer to ensure ratios of absorbance at 260 to 280 nm of 1.8–2.0. The reverse transcription was performed using the Transcriptor Reverse Transcriptase kit and random hexamer primers (Transcriptor RT; Roche Diagnostic, Mannheim, Germany). The RT-qPCR was performed using an ABI PRISMR 7300 Real-Time PCR System (Applied Biosystems, Foster City, CA, USA) and the FAM dye label format for the TaqMan Gene Expression Assays (Applied Biosystems, Foster City, CA, USA). The absolute values from each sample were normalized relative to the reference gene Beta2-microglobulin (*B2M)*. The relative quantification was calculated using the ΔΔCt method and normalized to the control group. Primers for the RT-qPCR were obtained based on the Applied Biosystems genome database of human mRNA references (Table S1).

### Animal and ethics statements

Male and female Wistar rats (Charles River Laboratories España S.A., Barcelona, Spain) weighing 200–250 g at the beginning of the experiments were maintained under a 12-h light/dark cycle in a humidity- and temperature-controlled room in the Animal Resource Center at the University of Málaga (Spain). Experiments and procedures were conducted under strict adherence to the European Directive 2010/63/EU on the protection of animals used for scientific purposes and the Spanish regulations for the care and use of laboratory animals (RD 53/2013 and 178/2004, Ley 32/2007 and 9/2003 and Decreto 320/2010). All efforts were made to reduce the number of animals and to minimize unnecessary pain and/or distress. All protocols and procedures were approved by the Ethic and Research Committee of the Universidad de Málaga (CEUMA).

### Cocaine treatments

Cocaine (Merck Life Science S.L.U., Madrid, Spain) was dissolved in sterile saline (0.9% NaCl). Cocaine or vehicle (saline) was administered by intraperitoneal (i.p.) injection in a volume of 1 mL/kg body weight.

#### Acute treatment

For acute treatment, male and female rats were administered with cocaine at different doses (5, 15 and 30 mg/kg) and animals were decapitated 30 and 240 min after cocaine or vehicle administration. Rats were randomly assigned to the different cocaine [dose 5 mg/kg: 30 min and 240 min (*n* = 14, 7 males and 7 females); dose 15 mg/kg: 30 min and 240 min (*n* = 14, 7 males and 7 females); dose 30 mg/kg: 30 min and 240 min (*n* = 14, 7 males and 7 females)] or vehicle [30 min and 240 min (*n* = 12, 6 males and 6 females)] conditions. Blood samples were collected and centrifuged (2000 × *g* for 15 min) to obtain plasma. Aliquots of plasma were stored at −80 °C for further analyses.

#### Chronic treatment

Male and female rats received an i.p. administration of cocaine (15 mg/kg) every day during 2 weeks (*n* = 16 per group, 8 males and 8 females). After the last injection, rats were decapitated at 2, 72 and 240 h, and blood samples were collected to obtain plasma, as previously described. Control rats received saline under the same conditions than cocaine groups.

### Determination of total LPA in plasma of rats

The concentration of total LPA in the plasma samples were measured using a commercial enzyme-linked immunosorbent assay (ELISA) kit following the manufacturer’s instructions (MyBioSource, San Diego, CA, USA). Data were expressed as nanograms of protein per milliliter of plasma (ng/mL).

### Statistical analysis

All clinical data in the tables are expressed as the number and percentage of subjects [N (%)], mean and standard deviation (mean ± SD) or median and interquartile range (median [IQR, 25–75%]). The significance of differences in the categorical and normal continuous variables was determined using Fisher’s exact test (chi-square test) and Student’s *t*-test (normal distribution) or Mann-Whitney *U* test (non-normal distribution), respectively. Multiple comparisons with raw data of LPA concentrations using the Mann-Whitney U test were corrected by controlling the false discovery rate (FDR) calculating corrected significance values (*q*-values) with the Benjamini-Hochberg procedure.

Two-way analysis of covariance (ANCOVA) was performed to indicate the main effects of two categorical independent variables of interest (i.e., “sex” and “diagnosis of CUD” factors) on the plasma concentrations of LPA species, while controlling for additional independent variables and covariates [e.g., age and BMI]. The *post hoc* tests for multiple comparisons were performed using Sidak’s correction test. Logarithm (10)-transformation for dependent variables was used to ensure statistical assumptions for positive skewed distributions and estimated marginal means [95 percent confidence intervals (95% CI)] of LPA species were expressed and represented in the figures after back transformations. Correlation analyses were performed using the Pearson’s coefficient (r) and multiple correlations were corrected by controlling the FDR calculating *q*-values with the Benjamini-Hochberg procedure.

Regarding rat studies, data in the graphs are expressed as mean and standard error of the mean (SEM). LPA concentrations were analyzed using one-way analysis of variance (ANOVA) followed by the Sidak’s *post hoc* test for multiple comparisons.

Tests statistic values and degrees of freedom were indicated in the results where appropriate. A *p* < 0.05 was considered statistically significant. The statistical analyses were carried out with the GraphPad Prism version 5.04 (GraphPad Software, San Diego, CA, USA), and IBM SPSS Statistical version 23 (IBM, Armonk, NY, USA).

## Results

### Sociodemographic and biological characteristics in the sample groups

Table 1 shows a sociodemographic and biological description of the sample. The median age of the CUD group was 35 years and the 83% of the participants were men with a median BMI of 25. Healthy controls were recruited with balanced sex composition and similar BMI to CUD group. However, the analysis of the total sample revealed significant differences in age between both groups, and patients in the CUD group were significantly younger than the control group (*p* < 0.001).

**Table 1:** Sociodemographic and biological characteristics of the study sample.

VARIABLE		Control group (*N* = 92)	CUD group (*N* = 105)	*p*-value
Age *Median (IQR)*	Years	38.0 (37–44)	35.0 (31–40)	**<0.001**^b^
BMI *Median (IQR)*	Kg/m^2^	24.6 (23–28)	25.3 (23–28)	0.925^b^
Sex *[N (%)]*	Women	20 (21.7)	18 (17.1)	0.471^a^
	Men	72 (78.3)	87 (82.9)	
Education degree *[N (%)]*	Elementary	3 (3.3)	19 (18.1)	**<0.001**^a^
	Secondary	50 (54.3)	73 (69.5)	
	University	39 (42.4)	13 (12.4)	

*p*-value in bold indicates a statistically significant difference.

*BMI* body mass index, *CUD* cocaine use disorders, *IQR* interquartile range.

^a^*p*-value was calculated using Fisher´s exact test or chi-square test.

^b^*p*-value was calculated using Man-Whitney test.

In addition, there were significant differences between control and CUD groups in education degree (*p* < 0.001).

### Plasma concentrations of LPA species in the sample groups

As shown in Table 2, raw data of the plasma LPA concentrations were compared in the total sample according to diagnosis of CUD and sex. Because LPA concentrations were not normally distributed, we used the median (IQR) and the Mann–Whitney *U* test.

**Table 2:** Raw data of the plasma concentrations of total LPA and LPA species according to the group and sex in the total sample.

Variable	Sample Group		*p*-value^a^	Sex		*p*-value^a^
	Control group (*N* = 92)	CUD group (*N* = 105)		Men (*N* = 159)	Women (*N* = 38)	
	*Median (IQR)*	*Median (IQR)*		*Median (IQR)*	*Median (IQR)*	
Total LPA (ng/mL)	144.48 (109.52–211.20)	114.52 (92.73–151.93)	**<0.001**	125.00 (96.79–164.50)	164.15 (120.13–231.97)	**0.002**
16:0 LPA (ng/mL)	20.53 (16.48–28.95)	18.95 (14.33–25.22)	**0.008**	19.04 (14.96–25.10))	25.22 (18.94–34.84)	**0.001**
18:0 LPA (ng/mL)	9.18 (7.85–10.27)	9.12 (7.48–10.32)	0.546^a^	8.77 (7.64–10.12)	9.80 (7.31–11.27)	0.288
18:1 LPA (ng/mL)	16.60 (9.51–20.6)	9.40 (7.72–12.07)	**<0.001**	9.93 (8.31–14.94)	13.10 (9.57–18.41)	**0.007**
18:2 LPA (ng/mL)	70.71 (51.03–104.49)	51.32 (35.71–76.65)	**<0.001**	54.69 (40.76–80.73)	84.63 (53.28–112.68)	**0.002**
20:4 LPA (ng/mL)	30.19 (22.29–47.55)	25.68 (21.28–39.37)	**0.011**	27.31 (22.12–38.70)	31.31 (21.57–50.48)	0.111

The corrected significance values (*q*-values) were calculated with the Benjamini Hochberg procedure (*q* = 0.0417 for “Sample Group”; and *q* = 0.0333 for “Sex”).

*p*-value in bold indicates a significant difference after correction.

*CUD* cocaine use disorder, *IQR* interquartile range, *LPA* lysophosphatidic acid.

^a^*p*-values from the Mann–Whitney *U* test.

We found that the patients with CUD had significantly lower concentrations of total LPA (*p* < 0.001), 16:0-LPA (*p* = 0.008), 18:1-LPA (*p* < 0.001), 18:2-LPA (*p* < 0.001) and 20:4-LPA (*p* = 0.011) than the control group. Regarding sex, women had significantly higher concentrations of total LPA (*p* = 0.002), 16:0-LPA (*p* = 0.001), 18:1-LPA (*p* = 0.007) and 18:2-LPA (*p* = 0.002) than men in the total sample.

### Plasma concentrations of LPA species in relation to diagnosis of CUD and sex

Log_10_-transformed data of plasma LPA concentrations were analyzed using two-way ANCOVA with “diagnosis of CUD” and “sex” as factors while controlling for age and BMI. Overall, there were no interaction effects between both factors, but there were significant main effects on some LPA species.

#### Age and BMI as covariates

Because there were significant differences in age when control and CUD groups were compared, we explored the association between LPA concentrations and age in the sample based on “diagnosis of CUD” (Table 3). Thus, there were significant and positive correlations between age and plasma concentrations of total LPA, 16:0-LPA, 18:1-LPA, 18:2-LPA, and 20:4-LPA in the total sample. When both groups were separately examined, we found similar significant positive associations between age and all of LPA species in the control group, but not in the CUD group.

**Table 3:** Correlation analyses between plasma concentrations of total LPA and LPA species with age and BMI.

Variables		Age *(years)*			BMI *(kg/m*^*2*^*)*		
		Control group	CUD group	Total sample	Control group	CUD group	Total sample
Total LPA (ng/mL)	r	+0.383	+0.160	+0.337	+0.013	+0.037	+0.019
	*p*-value	**<0.001**	0.103	**<0.001**	0.904	0.708	0.789
16:0-LPA (ng/mL)	r	+0.360	+0.172	+0.300	+0.057	+0.082	+0.067
	*p*-value	**<0.001**	0.080	**<0.001**	0.590	0.407	0.353
18:0-LPA (ng/mL)	r	+0.343	−0.063	+0.063	−0.059	+0.057	+0.022
	*p*-value	**0.001**	0.520	0.377	0.578	0.561	0.761
18:1-LPA (ng/mL)	r	+0.446	+0.177	+0.386	+0.012	−0.060	−0.025
	*p*-value	**<0.001**	0.072	**<0.001**	0.911	0.547	0.726
18:2-LPA (ng/mL)	r	+0.329	+0.112	+0.302	−0.026	+0.086	+0.022
	*p*-value	**0.001**	0.257	**<0.001**	0.806	0.385	0.760
20:4-LPA (ng/mL)	r	+0.404	+0.163	+0.305	+0.079	−0.057	−0.002
	*p*-value	**<0.001**	0.096	**<0.001**	0.452	0.565	0.976

Correlation analyses were performed using the Pearson’s coefficient (r).

The corrected significance values (*q*-values) were calculated with the Benjamini Hochberg procedure (*q* = 0.0306 for “Age”; and *q* = 0.0500 for “BMI”).

*p*-value in bold indicates a significant correlation after correction.

*BMI* body mass index, *CUD* cocaine use disorder, *LPA* lysophosphatidic acid.

Although there were no significant differences in BMI between both groups, we also explored the association between LPA concentrations and BMI in the sample based on “diagnosis of CUD” (Table 3). In contrast to age, we did not find any significant correlation between LPA concentrations and BMI in the different groups.

#### Plasma concentrations of LPA in controls and patients with CUD

**Fig. 1:**
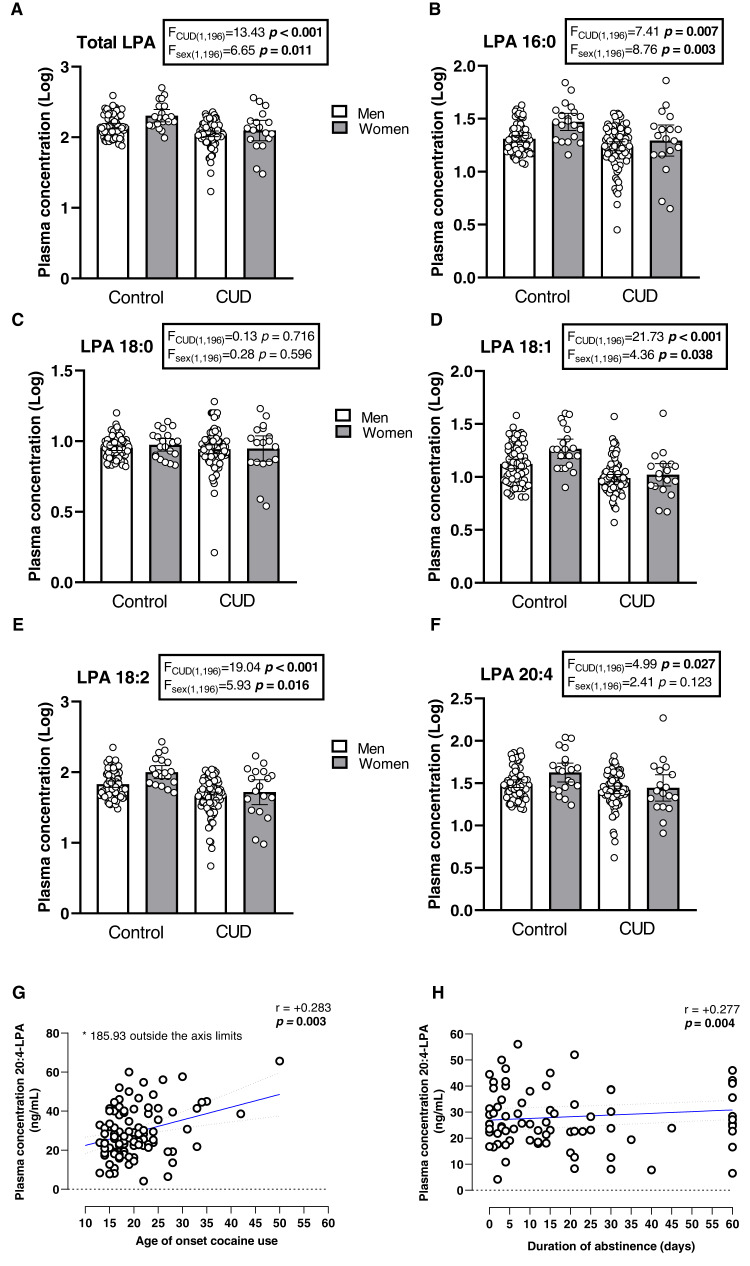
Plasma concentrations of total LPA and LPA species in the sample based on the diagnosis of CUD and sex. **A** Total LPA concentrations; (**B**) 16:0-LPA concentrations; (**C**) 18:0-LPA concentrations; (**D**) 18:1-LPA concentrations; (**E**) 18:2-LPA concentrations; and (**F**) 20:4-LPA concentrations. Dots are individual values. Bars represent the estimated marginal means and 95% CI of log10-transformed concentrations of total LPA and LPA species. Data were analyzed using two-way ANCOVA controlling for age and BMI. F-statistics and p-values of ANCOVA are shown. **G** Correlations between plasma concentrations of 20:4-LPA and age of onset cocaine use (years); and (**H**) duration of cocaine abstinence (days). Correlations analyses was performed using the Pearson’s correlation coefficient (r). Dots are individual values. Blue solid line and black dashed lines represent linear fit and 95% CI, respectively.

As shown in Fig. 1, the analysis revealed a main effect of “diagnosis of CUD” on plasma concentrations of total LPA (*F*_(1,196)_ = 13.43; *p* < 0.001), 16:0-LPA (*F*_(1,196)_ = 7.41; *p* = 0.007), 18:1-LPA (*F*_(1,196)_ = 21.73; *p* < 0.001), 18:2-LPA (*F*_(1,196)_ = 19.04; *p* < 0.001) and 20:4-LPA (*F*_(1,196)_ = 4.99; *p* = 0.027). Thus, there was a significant reduction in the concentration of total LPA and the majority of LPA species in the CUD group compared with the control group. However, there was no significant effect of “diagnosis of CUD” on 18:0-LPA concentrations (*F*_(1,196)_ = 0.13; *p* > 0.05).

#### Plasma concentrations of LPA in men and women

Regarding the influence of sex, the statistical analysis revealed a significant main effect of “sex” on plasma concentrations of total LPA (*F*_(1,196)_ = 6.65; *p* = 0.011), 16:0-LPA (*F*_(1,196)_ = 8.76; *p* = 0.003); 18:1-LPA (*F*_(1,196)_ = 4.36; *p* = 0.038); and 18:2-LPA (*F*_(1,196)_ = 5.93; *p* = 0.016). Specifically, these LPA species and total LPA were significantly higher in women than men (Fig. 1A–F).

### Clinical characteristics of the CUD group

The clinical characterization of the patients with CUD was assessed using relevant variables associated with the diagnosis of lifetime CUD (Table 4). The CUD group showed a median age of first use of cocaine of 18 years, with a median age of regular use onset of 25 years. This group displayed a median age of 5 years of regular cocaine use and 21 days of abstinence from this drug at the moment of the evaluation. Patients were diagnosed with a median of 8 DSM-IV-TR criteria for cocaine abuse and dependence, which indicates a severe CUD.

**Table 4:** Relevant cocaine-related variables, psychiatric comorbidity and medication in the CUD group.

Variable		CUD group (*N* = 105)
Age of first cocaine use *Median (IQR)*	*Years*	18 (16–22.5)
Age of starting regular cocaine use *Median (IQR)*	*Years*	25 (21–30.5)
Duration of regular cocaine use *Median (IQR)*	*Years*	5 (3–10.5)
DSM-IV-TR criteria for CUD *Median (IQR)*		8 (7–10)
Current cocaine abstinence *Median (IQR)*	*Days*	21 (4–60)
Comorbid substance use disorders *[N (%)]*	No	42 (40.0)
	Yes	63 (60.0)
	Alcohol	62 (59.0)
	Cannabis	23 (21.9)
	Sedatives	12 (11.4)
Comorbid psychiatric disorders *[N (%)]*	No	41 (39.0)
	Yes	64 (61.0)
	Mood disorders^a^	34 (32.4)
	Anxiety^b^	31 (29.5)
	Psychotic disorders^c^	8 (7.6)
	Borderline	32 (20.0)
	Antisocial disorders	23 (19.0)
Psychiatric medication use (last year) *[N (%)]*	No	32 (30.5)
	Yes	73 (69.5)
	Antidepressants	43 (41.0)
	Anxiolytics	52 (49.5)
	Anticraving	11 (10.5)
	Antipsychotics	11 (10.5)

*CUD* cocaine use disorders; *IQR* interquartile range.

^a^Major depressive episode, dysthymia, mania episode, hypomania episode and cyclothymia.

^b^Specific phobia, social phobia, panic disorder, agoraphobia, generalized anxiety disorder, obsessive compulsive disorder and posttraumatic stress disorder.

^c^Schizophrenia, schizoaffective, schizophreniform, delusional disorder, psychotic disorder not specified and brief disorder.

Regarding the prevalence of psychiatric comorbidity, we found that 60% of patients with CUD had comorbid SUD [mainly alcohol (59%) and cannabis (22%)] and 61% of these patients were diagnosed with comorbid mental disorders [mainly mood disorders (32%) and anxiety disorders (30%)]. In addition, 70% of the CUD group received psychiatric medication during the last year [mainly antidepressants (41%) and anxiolytics (50%)].

### Correlation analysis between plasma LPA species and variables related to CUD

We explored the association between the plasma concentrations of total LPA and LPA species, and variables related to CUD (i.e., age of first cocaine use, DSM criteria for CUD and length of cocaine abstinence). Notably, we only observed significant correlations of 20:4-LPA concentrations with both age of onset of cocaine use and length of cocaine abstinence (Table S2). Specifically, 20:4-LPA concentrations were positively correlated with age of first cocaine use (r = +0.283, *p* = 0.003) and length of cocaine abstinence (r = +0.277, *p* = 0.004) (Fig. 1G, H).

### mRNA expression of LPA receptors in the PBMCs of patients with CUD

**Fig. 2:**
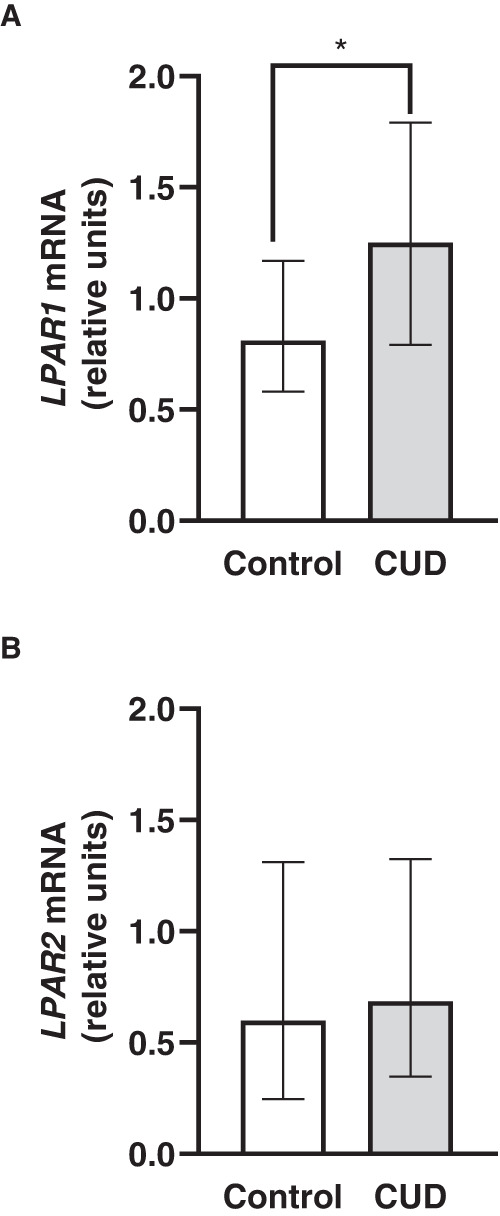
Relative mRNA expression levels of LPA receptor genes in the PBMC of healthy subjects and patients diagnosed with CUD. Relative mRNA expression of **A** LPA1; and **B** LPA2 receptors in the PBMC of patients diagnosed with CUD and healthy controls. Bars represent the median ± interquartile range (IQR). Data were analyzed using Mann–Whitney U test. (*) *p* < 0.05 denotes significant differences compared with the control group.

We also evaluated the effects of lifetime CUD on the mRNA expression levels of two main receptors involved in LPA signaling, LPA_1_ and LPA_2_ receptors, in the PBMCs of a representative sample of the control (*N* = 25) and the CUD (*N* = 63) groups (Fig. 2A, B). While the mRNA expression levels of LPA_1_ receptor in PBMCs for patients with CUD was significantly higher than that in healthy controls (*p* = 0.025), no significant differences were observed in the mRNA expression levels of LPA_2_ receptor between patients with CUD and controls. Furthermore, we also analyzed the correlation between the mRNA levels of LPA_1_ and LPA_2_ receptors and CUD-related variables (i.e., age of first cocaine use, DSM criteria for CUD and length of cocaine abstinence), and there were no significant associations (Table S3).

### Plasma concentrations of total LPA in rats exposed to cocaine

To further explore the impact of cocaine consumption on LPA concentrations, we performed a preclinical study using male and female Wistar rats exposed to acute and chronic cocaine administrations. As an end point, we decided to measure only total LPA concentrations since we found that this variable might reflect what we see in patients with CUD.

#### Rats exposed to acute cocaine at different doses

**Fig. 3:**
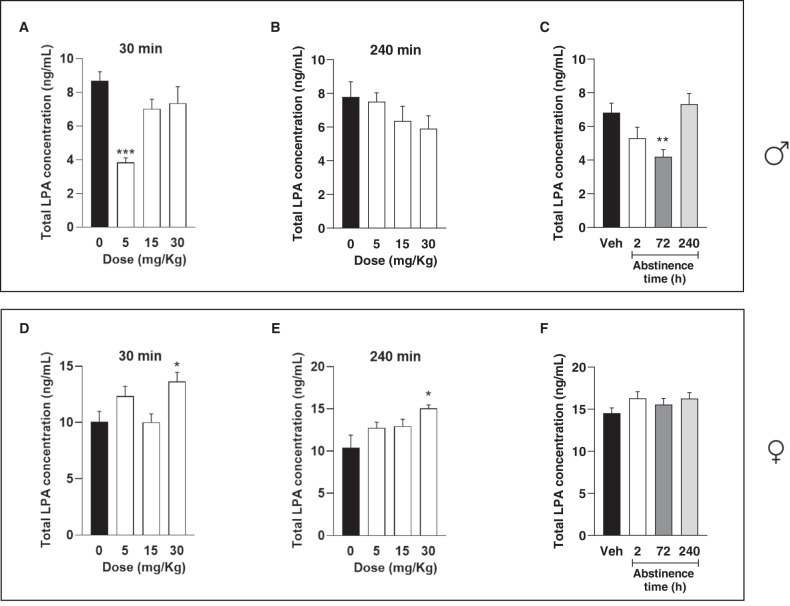
Plasma concentrations of total LPA in male and female Wistar rats exposed to cocaine. Plasma LPA concentrations in male rats after receiving an acute treatment with vehicle and different doses of cocaine at 30 min (**A**) and 240 min (**B**) after treatment. **C** Male rats received vehicle or 15 mg/kg of cocaine every day for 2 weeks and plasma concentrations of total LPA were determined at different time-points into abstinence. Plasma LPA concentrations in female rats after receiving an acute treatment with vehicle and different doses of cocaine at 30 min (**D**) and 240 min (**E**) after treatment. **F** Female rats received vehicle or 15 mg/kg of cocaine every day for 2 weeks and plasma concentrations of total LPA were determined at different time-points into abstinence. Bars represent the mean ± SEM. Data were analyzed using one-way analysis ANOVA followed by the Sidak’s post hoc test for multiple comparisons. (*) *p* < 0.05, (**) *p* < 0.01 and (***) *p* < 0.001 denote significant differences compared with vehicle-treated rats.

First, we tested whether total LPA concentrations were affected by the administration of different doses of cocaine (5, 15 and 30 mg/kg) in rats. In male rats, the analysis revealed a significant main effect of “dose of cocaine” on the plasma concentrations of total LPA (*F*_(3,23)_ = 9.81; *p* < 0.001) at 30 min after the treatment (Fig. 3A). The *post hoc* multiple comparisons showed that the male rats treated with 5 mg/kg displayed lower LPA concentrations (*p* < 0.001) than the vehicle group. However, we observed no significant differences (*F*_(3,23)_ = 1.32; *p* > 0.05) in the plasma concentrations of total LPA at 240 min after the cocaine treatment (Fig. 3B). In female rats, the analysis revealed a significant main effect of “dose of cocaine” on the plasma concentrations of total LPA (*F*_(3,23)_ = 4.60; *p* = 0.012) at 30 min after the treatment (Fig. 3D). The post hoc multiple comparisons showed that the female rats treated with 30 mg/kg displayed higher LPA concentrations (*p* < 0.05) than the vehicle group. Similar effects were observed at 240 min (Fig. 3E). Thus, the analysis revealed a significant main effect of “dose of cocaine” on the plasma concentrations of total LPA (*F*_(3,23)_ = 4.43; *p* = 0.014) at 240 min after the treatment, and the *post hoc* multiple comparisons showed that the female rats treated with 30 mg/kg displayed significantly higher LPA concentrations (*p* < 0.01) than the vehicle group.

#### Rats exposed to chronic cocaine

Because in humans we found that the 20:4-LPA concentrations were associated with length of cocaine abstinence, we investigated whether the LPA concentrations were also affected by duration of abstinence in male and female rats exposed to chronic cocaine. As shown in Fig. 3C, the analysis revealed a significant main effect of “duration of cocaine abstinence” on the plasma concentrations of total LPA (*F*_(3,28)_ = 6.23; *p* = 0.002) in male rats. In fact, groups of early abstinence displayed lower LPA concentrations than vehicle, being significant at 72 h of abstinence (*p* < 0.01), and reached the vehicle levels at 240 h. In contrast, we observed no significant effect of “duration of cocaine abstinence” on the plasma concentrations of total LPA (*F*_(3,28)_ = 1.36; *p* > 0.05.) in female rats (Fig. 3F).

## Discussion

The present study has examined the plasma concentration of LPA in men and women diagnosed with CUD, who were recruited from outpatient treatment programs for cocaine. We compared the LPA concentrations of patients with CUD with healthy controls, as well as the mRNA expression of LPA_1_ and LPA_2_ receptors in PBMCs of both groups. In addition, we performed a preclinical model of acute and chronic cocaine exposure in rats of both sexes to further explore the potential involvement of LPA signaling in cocaine addiction.

The main findings of the present study are summarized as follows: (1) patients with CUD had significantly lower plasma concentrations of total LPA and some LPA species than healthy subjects; (2) there were significant correlations between the plasma concentration of 20:4-LPA and relevant CUD-related variables (i.e., age of onset of cocaine use and duration of cocaine abstinence) and; (3) there were significant positive correlations between the plasma concentration of the different LPA species and age in the total sample and the control group, but not in the CUD group; (4) there were also sex differences in the expression of some species of LPA; (5) the CUD group had significantly higher mRNA expression of LPA_1_ receptor in PBMCs than healthy controls; and (6) rats exposed to cocaine displayed alterations on plasma LPA, and these alterations were dependent on sex, dose of cocaine and duration of the abstinence.

Our results showed that patients diagnosed with CUD displayed lower plasma concentrations of total LPA and the majority of LPA species than healthy subjects. These results are consistent with our previous studies in patients with AUD and patients with SUD [16–18]. However, we observed that the LPA species altered in each study were different, maybe due to the exposure to different substances (alcohol and/or cocaine). Thus, patients with CUD displayed alterations in plasma concentrations of 16:0-, 18:1-, 18:2-, and 20:4-LPA; patients with AUD displayed alterations in plasma concentrations of 16:0- and 18:1-LPA; and patients with SUD showed altered plasma concentrations of 16:0- and 18:2-LPA [17, 18]. These differences rise the question of the origin of the observed alterations. In addition to the effects of the diet on LPA species, these results suggest that cocaine exposure might be affecting fatty acid levels, and consequently, altering the fatty acid metabolism and the production of their derivatives. In this regard, we have previously described that patients with CUD have altered plasma concentrations of palmitoylethanolamide (PEA, 16:0-derived acylethanolamide), oleoylethanolamide (OEA, 18:1-derived acylethanolamide), linoleoylethanolamide (LEA, 18:2-derived acylethanolamide), and arachidonoylethanolamide and 2-arachidonoylglycerol (AEA and 2-AG, 20:4-derived acylethanolamide and acylglycerol, respectively), but not alterations in stearoylethanolamide concentrations (SEA, 18:0-derived acylethanolamide) [31]. Although these lipid mediators are produced at both central and peripheral levels, further research is necessary to determine whether cocaine affects plasma concentrations of LPA species through alterations at central and/or peripheral productions.

The exploration of the association between plasma LPA concentrations and CUD-related variables revealed a significant and positive correlation between age of onset of cocaine use and duration of cocaine abstinence and 20:4-LPA concentrations, a specie of LPA derived from arachidonic acid. Although the nature of these associations is unknown, one possible explanation might be that arachidonic acid is involved in many basic neuronal processes [32], and low levels of this polyunsaturated fatty acid are linked to an increased vulnerability to develop psychiatric disorders, including depressive disorders and SUD [33]. In fact, early age of cocaine use initiation is associated with higher severity of CUD and higher prevalence of comorbid SUD [34]. In the present study, the age of onset of cocaine use was before 20 years in 63% of the CUD group. We found that 59% of these patients with a younger age of initiation of cocaine use had comorbid SUD, and 59% of these were diagnosed with comorbid mental disorders. In addition, these patients were diagnosed with a median of 9 criteria for cocaine abuse and dependence, which indicates a severe CUD. Regarding the association between the plasma concentration of 20:4-LPA and duration of cocaine abstinence, a previous study has also described a correlation between the duration of alcohol abstinence and the 20:4-derived acylethanolamide AEA in patients diagnosed with AUD [35].

Because we found significant differences in age of the control and CUD groups, this physiological variable was controlled as a covariate in our statistical analysis. Moreover, our data revealed a significant association between the different LPA species and age in the total sample and in the control group. Previous studies in healthy subjects have reported the association between age and plasma LPA concentrations, although this influence is controversial [18, 36–38]. It is known that the LPA system is involved in the process of aging. Thus, it has been described the anti-aging and anti-oxidant effects of LPA [39]. Other reports have revealed the involvement of this system in resilience to ageing, and alterations of LPA signaling may be associated with late-life depression [40]. In the present study, we found no correlation between LPA concentrations and age in our cohort of patients with CUD, which suggests that the history of a pathological use of cocaine influenced the association of LPA concentrations and aging observed in the control group. This is concordant with the high prevalence of psychiatric comorbidity, mainly mood disorders, in our patients with CUD.

The present results also showed a sexual dimorphism in plasma LPA concentrations. Thus, women displayed higher plasma concentrations of LPA than men in the CUD and control groups. These results are in agreement with previous studies in healthy controls and patients with SUD and AUD [17, 18, 36, 37].

In the present study, we also evaluated the mRNA expression of LPA_1_ and LPA_2_ receptors in the PBMCs of controls and patients with CUD. Although both receptors activate similar downstream signaling pathways [22], at the present the LPA_1_ receptor has been only involved in drug addiction. Thus, studies in rodents have reported a main role of this receptor in cocaine addiction processes and alcohol-related behaviors [12–15]. In this regard, we did not find significant differences in the mRNA expression of LPA_2_ receptors between the CUD and control groups. However, we observed that patients with CUD displayed higher mRNA levels of LPA_1_ receptors than healthy controls. These alterations on the gene expression of these receptors in PBMCs support the role of LPA signaling system in CUD.

Although we had no sufficient sample to measure the different LPA species, we have assessed the plasma concentration of total LPA in male and female rats after acute and chronic cocaine treatment. Similar to the human data, these results in rats showed a sexual dimorphism in plasma LPA concentrations. Thus, female rats displayed higher plasma concentrations of total LPA than male rats treated with vehicle or cocaine. Although the present clinical results and other previous studies have reported sex differences in plasma concentrations of LPA [17, 18, 36, 37], we believe that this is the first study to assess the sexual dimorphism in LPA of animal models. Interestingly, after cocaine treatment in rats, we found significant changes depending on sex, dose of cocaine and duration of abstinence. In male rats, acute treatment with a low dose of cocaine reduced plasma concentrations of LPA in comparison with vehicle-treated rats, and these effects were only observed at 30 min. Conversely, the effects on LPA in female rats were opposite and longer than those observed in males. Thus, there was a significant increase in LPA concentrations in female rats treated with the highest dose of cocaine, and this effect was observed even at 240 min. Regarding chronic cocaine treatment, we found significantly lower LPA concentrations during early abstinence in male rats treated chronically with cocaine than vehicle-treated rats, although after several days of abstinence, cocaine-treated rats displayed similar LPA concentrations than did control rats. Again, we found a sexual dimorphism in these results, because we found no effects of duration of abstinence in female rats. Previous preclinical studies have reported sex differences in the response to cocaine in rats, and these differences might be associated to the influence of sex hormones, as well as differences in the brain of both male and female rats [41–46].

In conclusion, these findings support that lifetime CUD is associated with alterations in LPA signaling. Our results showed that LPA species and the expression of the LPA_1_ receptors are altered in patients diagnosed with CUD in comparison with healthy controls. In addition, plasma concentrations of 20:4-LPA displayed a significant association with age of onset of cocaine use and duration of abstinence. Moreover, LPA concentrations have shown a clear sexual dimorphism in the control and CUD groups. These data reveal the importance of monitoring alterations in this lipid signaling system, taking sex into account, to explore in future studies the role of LPA as reliable and valid biomarker for CUD to improve the stratification of men and women who demand treatment. In addition to these clinical findings, preclinical data also have shown alterations in plasma total LPA after both acute and chronic cocaine exposure, and these alterations were linked to sexual dimorphism.

Although these findings support the importance of monitoring LPA in the context of CUD, we are aware that this exploratory study has a number of limitations that future research should take into account. First, there were important statistical limitations related to the low number of women in comparison with men, mainly because women attend treatment programs to a lesser extent than men. It should be necessary to include large samples of women to confirm the sexual dimorphism observed in this study. Second, the recruitment of the sample was conducted from outpatient treatment programs, and there are many variables that are unknown (e.g., diet, physical activity, non-psychotropic medication, etc.) and that could influence the validity of the results. Third, we have not examined the protein expression of the LPA receptors in the PBMCs of participants. Because there are numerous posttranscriptional and translational mechanisms, the association between changes in the mRNA expression and the protein expression should be addressed in future research. Finally, future longitudinal studies will monitor changes in LPA species in the same patients considering different times of abstinence.

## Supplementary information

Table S1

Table S2

Table S3
