# Correction: Plasma concentrations of lysophosphatidic acid and the expression of its receptors in peripheral blood mononuclear cells are altered in patients with cocaine use disorders

**DOI:** 10.1038/s41398-024-03096-3

**Published:** 2024-09-17

**Authors:** María Flores-López, Nuria García-Marchena, Francisco J. Pavón-Morón, Nerea Requena-Ocaña, Laura Sánchez-Marín, Laura Martín-Chaves, Mónica García-Medina, Carmen Pedraza, Estela Castilla-Ortega, Juan J. Ruiz, Fernando Rodríguez de Fonseca, Pedro Araos, Antonia Serrano

**Affiliations:** 1 Instituto de Investigación Biomédica de Málaga y Plataforma en Nanomedicina (IBIMA-Plataforma BIONAND), 29590 Málaga, Spain; 2 Unidad de Gestión Clínica de Salud Mental, Hospital Regional Universitario de Málaga, 29010 Málaga, Spain; 3 Departamento de Psicobiología y Metodología de las Ciencias del Comportamiento, Facultad de Psicología, Universidad de Málaga, 29010 Málaga, Spain; 4 Unidad de Adicciones-Servicio de Medicina Interna, Institut d’Investigació en Ciències de la Salut Germans Trias i Pujol (IGTP), 08916 Badalona, Spain; 5 Unidad de Gestión Clínica Área del Corazón, Hospital Universitario Virgen de la Victoria de Málaga, 29010 Málaga, Spain; 6 Centro de Investigación Biomédica en Red de Enfermedades Cardiovasculares (CIBERCV), Instituto de Salud Carlos III, 28029 Madrid, Spain; 7 Centro Provincial de Drogodependencias de Málaga, Diputación Provincial de Málaga, 29010 Málaga, Spain; 8 Unidad de Gestión Clínica de Neurología, Hospital Regional Universitario de Málaga, 29010 Málaga, Spain

**Keywords:** Diagnostic markers, Addiction

Correction to: *Translational Psychiatry* 10.1038/s41398-023-02523-1, published online 21 June 2023

In this article the statement in the Funding information section was incorrectly given as ‘The present study has been supported by the following research programmes and projects: Redes de investigación Cooperativas Orientadas al Resultado en Salud (RECORs RD21/0009/0003) funded by Instituto de Salud Carlos III (ISCIII), Ministerio de Ciencia e Innovación and the European Regional Development Fund/European Social Fund (ERDF/ESF); Proyectos de Investigación en Salud (PI19/01577, PI19/00886 and PI20/01399) financed by ISCIII and ERDF/ESF; Proyecto de Investigación en Salud (PI-0140-2018) funded by Consejería de Salud y Familias, Junta de Andalucía, and ERDF/ESF. Proyectos I + D + I en el marco del Programa Operativo FEDER Andalucía 2014–2020 (UMA18-FEDERJA-059) funded by Consejería de Economía, Conocimiento, Empresas y Universidad, Junta de Andalucía and ERDF/ESF.MFL holds a pre-doctoral research contract (PFIS [F18/00249]) and NGM holds a ‘Sara Borrell’ Research Contract (CD19/00019) funded by ISCIII and ERDF-EU. FJP and AS hold a ‘Miguel Servet II’ Research Contract (CPII19/00022 and CPII19/00031, respectively) funded by ISCIII and ERDF-EU.’ and should have read, The present study has been supported by the following research programmes and projects: Redes de investigación Cooperativas Orientadas al Resultado en Salud (RECORs RD21/0009/0003) funded by Instituto de Salud Carlos III (ISCIII), Ministerio de Ciencia e Innovación and the European Regional Development Fund/European Social Fund (ERDF/ESF); Proyectos de Investigación en Salud ‘PI19/01577, PI19/00886 and PI20/01399’ funded by ISCIII and co-funded by the European Union; Proyecto de Investigación en Salud (PI-0140-2018) funded by Consejería de Salud y Familias, Junta de Andalucía, and ERDF/ESF. Proyectos I + D + I en el marco del Programa Operativo FEDER Andalucía 2014–2020 (UMA18-FEDERJA-059) funded by Consejería de Economía, Conocimiento, Empresas y Universidad, Junta de Andalucía and ERDF/ESF.MFL holds a pre-doctoral research contract (PFIS [F18/00249]) and NGM holds a ‘Sara Borrell’ Research Contract (CD19/00019) funded by ISCIII and ERDF-EU. FJP and AS hold a ‘Miguel Servet II’ Research Contract (CPII19/00022 and CPII19/00031, respectively) funded by ISCIII and ERDF-EU.’.

The original article has been corrected.

