# Supplementary material for: Plasma concentrations of lysophosphatidic acid and the expression of its receptors in peripheral blood mononuclear cells are altered in patients with cocaine use disorders
Source: Transl Psychiatry. 2023 Jun 21;13:215. doi: 10.1038/s41398-023-02523-1 (PMC10284796; doi:10.1038/s41398-023-02523-1)
Supplement: Supplementary file 1 — Table S1 [file 41398_2023_2523_MOESM1_ESM.docx]

**Table S1.** Primer references for TaqMan® Gene Expression Assays (Applied Biosystems).

| GENE DESCRIPTION | Assay ID | RefSeq | Amplicon Length |
| --- | --- | --- | --- |
| *B2M* | Hs00187842_m1 | [NM_004048.2](http://www.ncbi.nlm.nih.gov/nuccore/NM_004048.2) | 64 |
| *LPAR1* | Hs00173500_m1 | [NM_005577.2](http://www.ncbi.nlm.nih.gov/nuccore/NM_005577.2) | 89 |
| *LPAR2* | Hs01109356_m1 | [NM_004720.5](http://www.ncbi.nlm.nih.gov/nuccore/NM_004720.5) | 83 |

Abbreviations: *B2M* = Beta2-microglobulin; *LPAR1* = LPA_1_ receptors; *LPAR2* = LPA_2_ receptors; RefSeq = reference sequences
