# Supplementary material for: Plasma concentrations of lysophosphatidic acid and the expression of its receptors in peripheral blood mononuclear cells are altered in patients with cocaine use disorders
Source: Transl Psychiatry. 2023 Jun 21;13:215. doi: 10.1038/s41398-023-02523-1 (PMC10284796; doi:10.1038/s41398-023-02523-1)
Supplement: Supplementary file 2 — Table S2 [file 41398_2023_2523_MOESM2_ESM.docx]

**Table S2.** Correlation analyses between plasma concentrations of total LPA and LPA species with the age of first cocaine use and the length of cocaine abstinence.

| VARIABLES | | CUD group  (*N* = 105) | |
| --- | --- | --- | --- |
|  |  | **Age of onset cocaine use**  *(years)* | **Duration of cocaine abstinence**  *(days)* |
| Total LPA  (ng/mL) | r | +0.175 | +0.094 |
|  | *p*-value | 0.075 | 0.340 |
| 16:0-LPA  (ng/mL) | r | +0.155 | -0.005 |
|  | *p*-value | 0.114 | 0.957 |
| 18:0-LPA  (ng/mL) | r | +0.014 | +0.064 |
|  | *p*-value | 0.890 | 0.518 |
| 18:1-LPA  (ng/mL) | r | +0.169 | -0.001 |
|  | *p*-value | 0.084 | 0.989 |
| 18:2-LPA  (ng/mL) | r | +0.059 | -0.012 |
|  | *p*-value | 0.549 | 0.903 |
| 20:4-LPA  (ng/mL) | r | +0.283 | +0.277 |
|  | *p*-value | **0.003** | **0.004** |

Correlation analyses were performed using the Pearson’s coefficient (r).

The corrected significance values (*q*-values) were calculated with the Benjamini Hochberg procedure (*q* = 0.0083 for “Age of onset cocaine use”; and *q* = 0.0083 for “Duration of cocaine abstinence”).

*p*-value in bold indicates a significant correlation after correction.

Abbreviations: CUD = cocaine use disorder; LPA = lysophosphatidic acid.
