# Supplementary material for: Plasma concentrations of lysophosphatidic acid and the expression of its receptors in peripheral blood mononuclear cells are altered in patients with cocaine use disorders
Source: Transl Psychiatry. 2023 Jun 21;13:215. doi: 10.1038/s41398-023-02523-1 (PMC10284796; doi:10.1038/s41398-023-02523-1)
Supplement: Supplementary file 3 — Table S3 [file 41398_2023_2523_MOESM3_ESM.docx]

**Table S3.** Correlation between mRNA levels of LPA_1_ and LPA_2_ receptors with CUD-related variables.

| VARIABLE | Age of onset cocaine use  (*N* = 63) | DSM-IV-TR cocaine criteria  (*N* = 63) | Duration of cocaine abstinence  (*N* = 63) |
| --- | --- | --- | --- |
|  | **r** | **r** | **r** |
|  | ***p*-value** | ***p*-value** | ***p*-value** |
| *LPAR1* | +0.031 | -0.129 | +0.033 |
|  | 0.811 | 0.312 | 0.800 |
| *LPAR2* | -0.034 | -0.041 | +0.094 |
|  | 0.792 | 0.748 | 0.458 |

Correlation analyses were performed using the Pearson’s coefficient (r).

Abbreviations: r= Pearson’s correlation coefficient
